# Oreoch-1: A Peptide from *Oreochromis niloticus* as a Potential Tool against Staphylococci

**DOI:** 10.3390/pathogens12101188

**Published:** 2023-09-23

**Authors:** Francesca Palma, Annalisa Chianese, Erica Panico, Giuseppe Greco, Alessandra Fusco, Vittoria Savio, Eleonora Ruocco, Alessandra Monti, Nunzianna Doti, Carla Zannella, Giovanna Donnarumma, Anna De Filippis, Massimiliano Galdiero

**Affiliations:** 1 Department of Experimental Medicine, University of Campania “Luigi Vanvitelli”, 80138 Naples, Italy; francesca.palma@unicampania.it (F.P.); annalisa.chianese@unicampania.it (A.C.); alessandra.fusco@unicampania.it (A.F.); vittoriasavio@libero.it (V.S.); carla.zannella@unicampania.it (C.Z.); giovanna.donnarumma@unicampania.it (G.D.); anna.defilippis@unicampania.it (A.D.F.); 2 UOC of Virology and Microbiology, University Hospital of Campania “Luigi Vanvitelli”, 80138 Naples, Italy; erica.panico@policliniconapoli.it (E.P.); giuseppe.greco@unicampania.it (G.G.); 3 Dermatology Unit, Department of Mental and Physical Health and Preventive Medicine, University of Campania “Luigi Vanvitelli”, 80138 Naples, Italy; eleonora.ruocco@unicampania.it; 4 Institute of Biostructures and Bioimaging (IBB), National Research Council (CNR), 80131 Naples, Italy; alessandra.monti@ibb.cnr.it (A.M.); nunzianna.doti@cnr.it (N.D.)

**Keywords:** antimicrobial resistance, *Staphylococcus aureus*, *Staphylococcus epidermidis*, biofilm, antimicrobial peptide

## Abstract

Staphylococci, including *Staphylococcus aureus* and *Staphylococcus epidermidis*, are important human pathogens associated with potentially life-threatening infections. Their great biofilm-producing ability and the development of resistance mechanisms often account for therapeutic failure. Hence, the scientific community has devoted intensive efforts to the development of antimicrobial compounds active against both planktonic and sessile bacterial populations. Contextually, antimicrobial peptides (AMPs) are natural peptides produced by the innate immunity of every organism, representing a potential new therapeutic solution against human microbial pathogens. Our work focused on the in vitro activity of Oreoch-1, an AMP from the gills of Nile tilapia (*Oreochromis niloticus*), against standard and clinical *S. aureus* and *S. epidermidis* strains. Firstly, the cytotoxicity profile of Oreoch-1 was determined in human colon carcinoma cells. Secondly, its antibacterial spectrum was explored against staphylococcal strains to set up the minimum inhibitory concentration (MIC) and the minimum bactericidal concentration (MBC). Our results highlighted an antibacterial activity in the range 6.25–25 μM, with a general bacteriostatic effect. Therefore, the biofilm-inhibitory property was assessed against *S. aureus* ATCC 25923 and *S. epidermidis* ATCC 35984, indicating a significant reduction in *S. aureus* biomass at sub-MIC concentrations. Overall, our study indicates Oreoch-1 as a promising new therapeutic weapon against staphylococcal infections.

## 1. Introduction

Antimicrobial resistance (AMR) and biofilm tolerance are the main factors involved in the persistence of bacterial infections. In recent years, there has been an alarming reduction in the efficacy of antibiotic treatments related to a growing resistance acquired by pathogens [1]. The discovery of new antimicrobials is an ever-increasing challenge in clinical environments and in global health care. Therefore, there is an urgent need for innovative medicines to counteract this phenomenon.

*Staphylococcus aureus* (*S. aureus)* and *Staphylococcus epidermidis* (*S. epidermidis*) are Gram-positive bacteria and major pathogens in humans and animals, causing a wide variety of diseases ranging from skin and soft tissue infections to life-threatening invasive diseases. *S. epidermidis* belongs to the group of coagulase-negative staphylococci (CoNS), which, unlike coagulase-positive staphylococci, such as *S. aureus*, lacks the coagulase enzyme [2]. *S. aureus* together with *S. epidermidis* are among the major causes of infections related to commensal agents. They are also responsible for the colonization of medical implants and nosocomial infections worldwide [3]. *S. aureus* colonizes about 30% of the human population; simultaneously, it is a leading cause of bacteremia, infective endocarditis, and skin, soft tissue, osteoarticular, and device-related infections [4]. Its ability to infect and develop biofilm on prosthetic devices within the host is a well-established virulent trait, making patient management more challenging [5,6]. Biofilm is a complex community of sessile bacteria encased in a dense extracellular matrix of water, nutrients, polysaccharides, DNA, and proteins which provides protection from the immune system and drugs, hence significantly limiting the available treatment options [7]. According to a report released in 2022 by The European Center for Disease Prevention and Control (ECDC), *S. aureus* was the second most isolated species in the European area in 2020, and more than half of Italian isolates were resistant to methicillin [8]. Hence, the increasing trend of antibiotic resistance, along with the biofilm-, cytotoxins-, and superantigens-producing ability, classifies this organism as a major human pathogen, associated with a high burden in terms of morbidity and mortality [4]. By contrast, the colonization of human and animal skin and mucous membranes is the main source of endogenous infections by *S. epidermidis* [3]. Although CoNS are generally equipped with fewer virulence properties than *S. aureus*, *S. epidermidis* is a very common cause of nosocomial bacteremia, with a high invasive capability particularly in preterm newborns and in neutropenic cancer patients [3,9,10]. Furthermore, it can efficiently inhabit biomaterial surfaces, forming biofilm and establishing a persistent infection. The isolation of methicillin-resistant *S. epidermidis*, as well as strains poorly susceptible to aminoglycosides, macrolides, and vancomycin, has been reported worldwide [9,11,12]. Therefore, considering the relevant clinical burden of *S. aureus* and *S. epidermidis*, there is an urgent need for developing alternative antimicrobial agents.

Antimicrobial peptides (AMPs) are the host defense peptides of the innate immune systems of every form of life [13]. Their broad-spectrum action against bacteria, fungi, and viruses has been widely reported so far [11,13,14,15,16]. The cationic nature of the majority of AMPs accounts for their negatively charged interaction with bacterial surfaces and for their selectivity towards neutral-charged mammalian membranes [14]. Interestingly, the microbial membrane impairment due to the antimicrobial peptide interaction is less likely associated with resistance development, compared to common antibiotics. In light of these considerations, the current study focused on the in vitro antistaphylococcal properties exerted by Oreoch-1, an AMP named oreochromicin isolated from Nile tilapia (*Oreochromis niloticus*). Specifically, in this study, we evaluated the activity of an Oreoch-1 analog amidated at the C-terminal end to obtain a more positively charged sequence compared to the natural one, in order to positively modulate the interaction with the bacterial cell membrane [17]. Our interesting results against the planktonic form of *S. aureus* and *S. epidermidis*, and against the sessile counterpart of *S. aureus*, present Oreoch-1 as a potential new antimicrobial peptide to deal with staphylococcal infections.

## 2. Materials and Methods

### 2.1. Peptide Synthesis and Characterization

**Figure 1:**
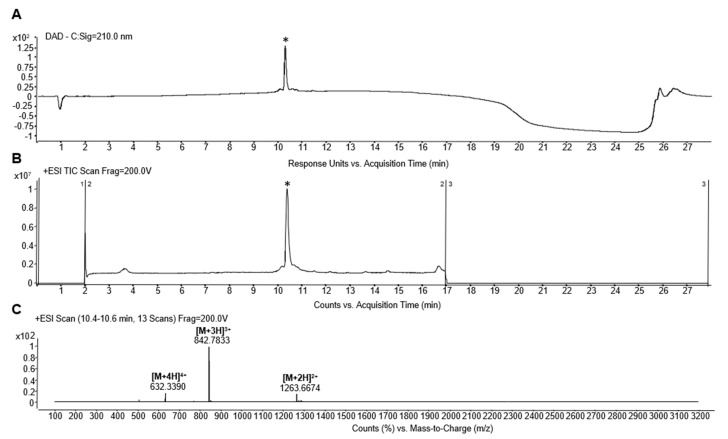
HPLC (**A**,**B**) and MS (**C**) profiles of purified Oreoch-1. The retention time (tR) value of the target peptide was about 10.4 min (**A**,**B**). (**C**) MS analysis showed the expected mass for Oreoch-1 at m/z: 1263.667, [M+2H]^+^; 842.783, [M+3H]^2+^; 632.339, [M+4H]^3+^. The experimental MW of 2525.36 Da coincides with the theoretical monoisotopic mass 2525.39 Da. (*) indicates the chromatographic peak containing the target peptide.

The C-terminally amidated peptide Oreoch-1 (single letter sequence: FIHHIIGGLFSVGKHIHGLIHGH-NH_2_) was synthesized using oxyma/DIC as coupling agents, and following methods reported in the literature [18]. The HPLC preparative purification was carried out on a WATERS 2545 preparative system (Waters, Milan, Italy) fitted out with a WATERS 2489 UV/Visible detector, applying a linear gradient of CH_3_CN/0.05%TFA in water 0.05% TFA from 5 to 70% in 20 min (min), at a flow rate of 12 mL/min. MS characterization of the peptide was performed using an ESI-TOF-MS Agilent 1290 Infinity LC System coupled to an Agilent 6230 time-of-flight (TOF) LC/MS System (Agilent Technologies, Cernusco sul Naviglio, Italy). The LC Agilent 1290 LC module was coupled with a photodiode array (PDA) detector and a 6230 time-of-flight MS detector, along with a binary solvent pump degasser, a column heater, and an autosampler. LC-MS characterization of the peptide was performed using a C18 Waters xBridge column (3 μm, 4.6 × 5.0 mm), applying a linear gradient of CH_3_CN/0.05%TFA in water 0.05% TFA from 5 to 70% in 20 min, at a flow rate of 0.2 mL/min. The yields of the target peptides were calculated as [(experimental weight of pure peptide)/(theoretical weight) × 100], where the theoretical weight was calculated on the basis of the synthesis scale used, and estimated to be about 80%. The relative purity of peptides was calculated as the ratio of the peak area of the target peptide and the sum of areas of all detected peaks from the UV chromatograms at 210 nm (Figure 1). The purity of the peptide was estimated to be greater than 95%. Protected amino acids, coupling agents (HATU, Oxyma), Fmoc-Rink Amide AM resin used for peptide synthesis, and solvents, including acetonitrile (CH_3_CN), dimethylformamide (DMF), and other products such as trifluoroacetic acid (TFA), sym-collidine, diisopropylethylamine (DIPEA), and piperidine, were purchased from Merck (Milan, Italy). The peptide was solubilized in sterile water and stored at −20 °C until use. 

### 2.2. In Vitro Cytotoxicity Evaluation

Oreoch-1 cytotoxicity was tested using a (3-(4,5-dimethylthiazol-2-yl)-2,5-diphenyltetrazolium bromide (MTT)) assay on human colorectal adenocarcinoma (Caco-2) cells, purchased from the American Type Culture Collection (ATCC, Manassas, VA, USA). In detail, cells (3.5 × 10^4^/well), routinely grown in Dulbecco’s Modified Eagle Medium (DMEM) and supplemented with 1% antibiotic solution, 1% L-Glutamine, and 10% Fetal Bovine Serum (FBS) (Microgem, Pozzuoli, Italy), were seeded in a 96-multiwell plate and incubated at 37 °C in a humified atmosphere with 5% CO_2_. The following day, the adherent cell monolayer was treated with decreasing peptide concentrations (200–1.56 μM), and resuspended in water. The untreated cells and cells exposed to 100% dimethyl sulfoxide (DMSO) were considered as negative (CTR−) and positive (CTR+) controls, respectively. After 20 h (h) of treatment, the cell viability was determined using a 5 mg/mL MTT solution (Sigma-Aldrich, St. Louis, MO, USA), by dispensing 100 μL in each well. After 3 h of incubation at 37 °C, the formazan crystals were dissolved with pure DMSO; then, the absorbance was read at 570 nm using a microplate reader (Tecan, Männedorf, Switzerland). In the end, the percentage of cytotoxicity was calculated according to the following formula:

% Cytotoxicity = [1 − (Abs_570 nm_ of the test sample)/Abs_570 nm_ of CTR −) × 100

### 2.3. Microorganisms: Isolation, Drug Susceptibility, and Culture Conditions

Standard strains of *S. aureus* (ATCC 6538 and ATCC 25923) and of *S. epidermidis* (ATCC 12228 and 35984) were purchased from ATCC. For both bacterial species, clinical strains were collected from different human specimens and kindly provided by the University Hospital of Campania “Luigi Vanvitelli” (Naples, Italy). Specifically, the isolates were plated on Columbia CNA Agar with 5% Sheep Blood (BioMerieux, Marcy-l’Étoile, France) and incubated overnight at 37 °C. Bacterial identification and susceptibility tests were worked out using Matrix-Assisted Laser Desorption/Ionization–Time of Flight (MALDI-TOF, Bruker Daltonics, Bremen, Germany) and the Phoenix BD system (Becton Dickinson, Franklin Lakes, NJ, USA), respectively, as previously described [19,20]. After 16 h of incubation, the resistance patterns were interpreted according to the EUCAST breakpoints. Table 1 lists the features of the bacterial strains exploited during this study. Before any antibacterial tests, every strain was streaked on Brain Heart Infusion (BHI) Agar (Sigma-Aldrich, St. Louis, MO, USA) and incubated at 37 °C for 18 h.

### 2.4. Antibacterial Assay

The antibacterial potential of Oreoch-1 was assessed against reference and clinical strains. The minimum concentration able to inhibit 80% of bacterial growth (MIC_80_) and the minimum bactericidal concentration (MBC) were deduced by exploiting the broth microdilution method. The activity of the peptide was checked in the concentration range of 25–1.56 μM. Briefly, a pure colony from BHI-agar was inoculated in BHI broth and incubated at 37 °C under shaking conditions (180 rpm), until the mid-log phase. Hence, a bacterial suspension of 1 × 10^6^ colony-forming units/mL (CFU/mL) in fresh BHI broth was set up and dispensed (50 μL/well) in a microtiter plate, previously filled with decreasing concentrations of Oreoch-1 (final density of 5 × 10^5^ CFU/well). Vancomycin was tested at 2 and 4 μg/mL against *S. aureus* and *S. epidermidis* strains, respectively, as CTR+, whilst untreated staphylococcal cells constituted the CTR−. After 20 h incubation at 37 °C, bacterial turbidity was measured spectrophotometrically at 600 nm. Then, the rate of growth inhibition was determined via the following formula:

% Growth Inhibition = [1 − (Abs_600 nm_ of the test sample)/Abs_600 nm_ of CTR−] × 100

In the end, to obtain the MBC values, aliquots (50 μL) from wells without evident bacterial growth were spotted on BHI agar plates and incubated at 37 °C for 20 h. MBC refers to the lowest peptide concentration causing a 99.9% reduction in the initial bacterial population.

### 2.5. Biofilm Maturation Test

The potential inhibitory effect of Oreoch-1 during the biofilm maturation phase was explored against *S. aureus* ATCC 25923 and *S. epidermidis* ATCC 35984, chosen as biofilm-producer standard strains. In detail, in a 96-multiwell plate, 100 μL from a bacterial suspension (2 × 10^8^ CFU/mL) in Luria–Bertani (LB) broth (Sigma-Aldrich, St. Louis, MO, USA) supplemented with 1% glucose was challenged with an equal volume of the peptide under analysis and tested in the range 25–1.56 μM. The treatment was carried out for 20 h at 37 °C under static conditions. Then, floating cells were discarded, and the mature biofilm was washed twice with Phosphate-Buffered Saline 1× (PBS, Microgem, Pozzuoly, Italy), air-dried, and stained with 0.05% crystal violet (CV) solution for 40 min under slight shaking for dying homogenization. The plate was rinsed with distilled water and air-dried. In the end, CV was dissolved with 100% ethanol and the absorbance of the stained staphylococcal biofilm was recorded at 570 nm with a multiwell reader (Tecan, Männedorf, Swiss). Biofilms formed by bacteria that did not undergo any treatment with Oreoch were used as negative controls for comparison with the treated counterpart following the formula:

% Biofilm Inhibition = [1 − (OD_570 nm_ of the test sample/OD_570 nm_ of CTR−)] × 100

Furthermore, the same experiment was performed to assess the number of viable sessile cells. Specifically, the 20 h mature biofilm, obtained as described, was washed twice with PBS 1× to remove planktonic cells, and resuspended after vigorous cracking in PBS 1×. Then, serial 10-fold dilutions were spotted on BHI agar plates and incubated at 37 °C. The following day, grown colonies were counted to define CFU/mL from the Oreoch-treated and untreated biofilm.

### 2.6. Biofilm Evaluation by Fluorescence Microscopy

Biofilm formation was analyzed using the FilmTracer^TM^ LIVE/DEAD^®^ Biofilm Viability Kit (Invitrogen, Waltham, MA, USA) containing a mixture of SYTO 9 dye (3.34 mM) and propidium iodide (PI, 20 mM). Shortly after, in a 6-well plate, the mature biofilm, exposed to 1×MIC and ½×MIC values, was stained with the mixture. Biofilm developed in the absence of any drug constituted the CTR−. After 30 min of staining in the dark at room temperature (RT), fluorescent images were collected using a Nikon Ti2-U research inverted fluorescence microscope (Nikon Instruments, Amsterdam, The Netherlands) with beam path settings for FITC and TRITC-like labels. In detail, the stain differentiates live bacteria, appearing fluorescent green, from dead cells releasing fluorescent red, while the background remains virtually nonfluorescent. Stacks of about 20 images were collected using a 20× objective lens.

### 2.7. Statistical Analysis

All the experiments were carried out in technical and biological triplicate, and data were expressed as mean ± standard deviation (SD). MIC_80_ and the cytotoxic concentration of 50% (CC_50_) values were calculated using the GraphPad Software Prism 10 (San Diego, CA, USA). The significance of the difference between treated samples and the untreated counterpart was obtained with Dunnett’s test using the same software. The *p*-value < 0.05 was considered significant. 

## 3. Results

### 3.1. Evaluation of Cytotoxicity Profile

**Figure 2:**
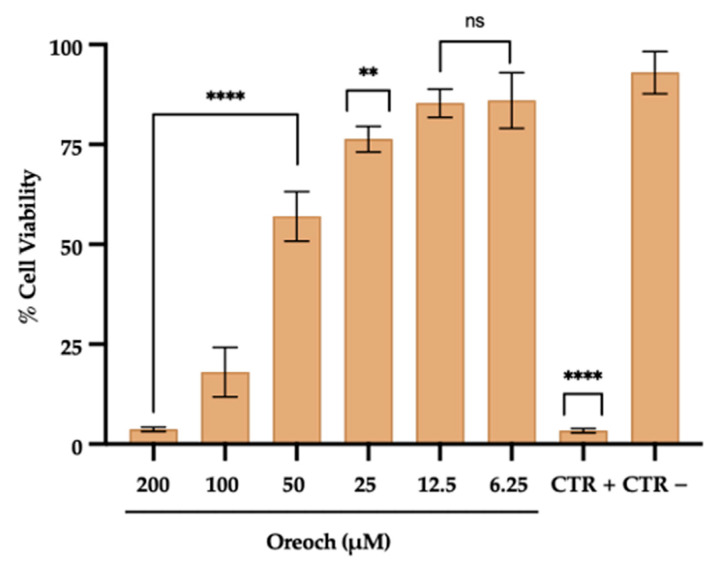
Cytotoxicity profile of Oreoch-1 assessed on Caco-2 cell line. The data represent the mean ± SD. CTR−: untreated cells; CTR+: cells exposed to 100% DMSO. Dunnett’s multiple comparisons tests: **** *p*-value < 0.001; ** *p*-value = 0.0029; ns = not significant.

Caco-2 cells, treated with different concentrations of Oreoch-1, were exploited as a model to assess its in vitro cytotoxic effect (Figure 2). The peptide decreased cell viability in a concentration-dependent manner. In detail, Oreoch-1 significantly affected cell viability in the range 200–50 μM (*p*-value < 0.0001) within 20 h. In contrast, the monolayer exposed to 25 μM of peptide registered a viability rate of 77%, and no relevant cell damage was detected at lower dosages when compared to the untreated counterpart (CTR−). The treatment of Caco-2 cells with 100% DMSO, used as CTR+, resulted in a cell mortality rate of 97%. The concentration able to induce 50% of cell death (CC_50_) was 59.53 μM. 

### 3.2. Evaluation of Antibacterial Effect

**Figure 3:**
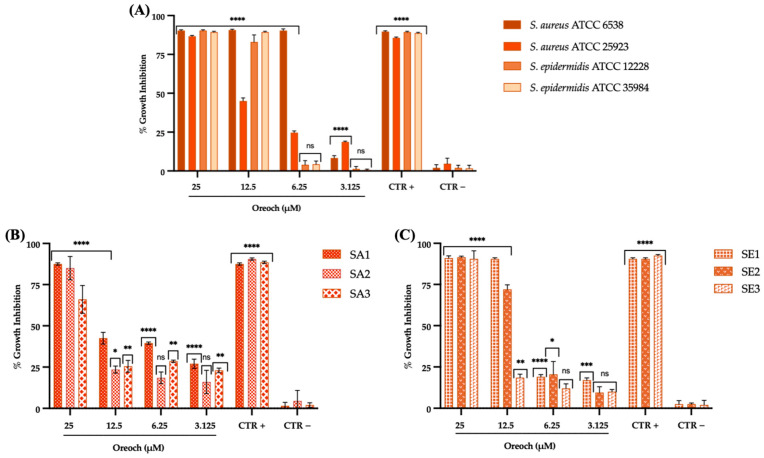
Staphylococcal growth inhibition by Oreoch-1 after 20 h of treatment. Bacterial cells from ATCC (**A**), *S. aureus* (**B**), and *S. epidermidis* (**C**) clinical strains challenged with vancomycin (2 and 4 μg/mL for *S. aureus* and *S. epidermidis*, respectively), and the untreated counterpart accounted for CTR+ and CTR−, respectively. The data represent the mean ± SD. Dunnett’s multiple comparisons tests: (**A**) **** *p*-value < 0.001; ns = not significant; (**B**) **** *p*-value < 0.0001; ** *p*-value < 0.0080; * *p*-value = 0.0490; ns = not significant; (**C**) **** *p*-value < 0.0001; *** *p*-value = 0.0002; ** *p*-value = 0.0053; * *p*-value = 0.0141; ns = not significant.

Oreoch-1 displayed antibacterial activity against standard, as well as clinical, drug-resistant *S. aureus* and *S. epidermidis* strains. The first evaluation allowed the determination of MIC and MBC of the peptide for the studied microorganisms. A greater susceptibility for *S. aureus* ATCC 6538 to the peptide was observed, if compared to the other tested standard bacteria (Figure 3). Indeed, the MIC_80_ recorded for this strain (6.25 μM) demonstrated to be two-fold lower than biofilm-negative and -positive *S. epidermidis* cells (12.5 μM) and four-fold lower than the MIC_80_ value of biofilm-producer *S. aureus* (25 μM). The trend followed by clinical strains was more heterogenous, as expected from analyzing their antibiotic resistance pattern. In detail, the least susceptible isolate, impaired only by 66% at the highest Oreoch-1 concentration, was SA3; for SA1-2 and SE2-3, the MIC_80_ value was registered at 25 μM, whereas SE1 emerged as the most sensitive clinical strain to the treatment (MIC_80_ = 12.5 μM). The MBC evaluation revealed a general bacteriostatic effect, except for *S. aureus* ATCC 6538, whose bacterial load was reduced by 99.99% by Oreoch-1 at 2×MIC. 

### 3.3. Antibiofilm Action Analyzed via Fluorescence Microscopy

**Figure 4:**
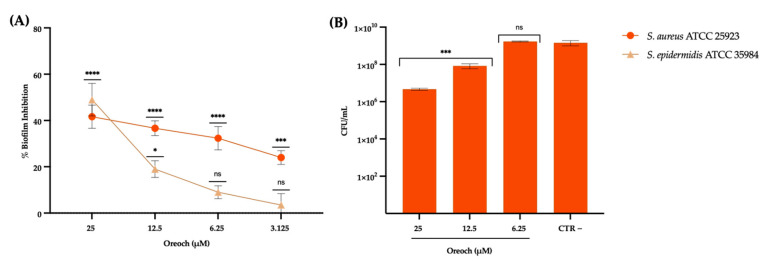
Effect of Oreoch-1 against sessile population of *S. aureus* and *S. epidermidis*, evaluated with crystal violet staining (**A**), and *S. aureus* viable sessile cell count (**B**), after the employment of the peptide. Unexposed sessile bacteria accounted for CTR−. The data represent the mean ± SD. Dunnett’s multiple comparisons tests: (**A**) **** *p*-value < 0.001; *** *p*-value = 0.0001; * *p*-value = 0.0248; ns = not significant; (**B**) *** *p*-value < 0.0003; ns = not significant.

The results of the biofilm production, when bacteria were treated with different dosages of Oreoch-1, are reported in Figure 4, and expressed in the biofilm impairment rate compared to the untreated control (CTR−). According to the CV staining data, 1×MIC and sub-MIC concentrations (1/2-1/8×MIC) of the peptide inhibited the biofilm formation in *S. aureus* ATCC 25923 in a dose-dependent manner, with an inhibitory activity rate of 41.6 and 36.6% at 25 and 12.5 μM, respectively. On the other hand, the impairment of *S. epidermidis* ATCC 35984 biofilm was weaker (19%) at 1×MIC value, and from sub-MIC concentrations, it was not significantly affected. Hence, *S. aureus* was selected for further investigations. The counting of viable sessile cells highlighted that biofilm-associated bacteria remarkably decreased after the exposure to 25 and 12.5 μM (*p*-value < 0.0003), whereas at 6.25 μM, no significant difference was observed if compared to CTR−. 

**Figure 5:**
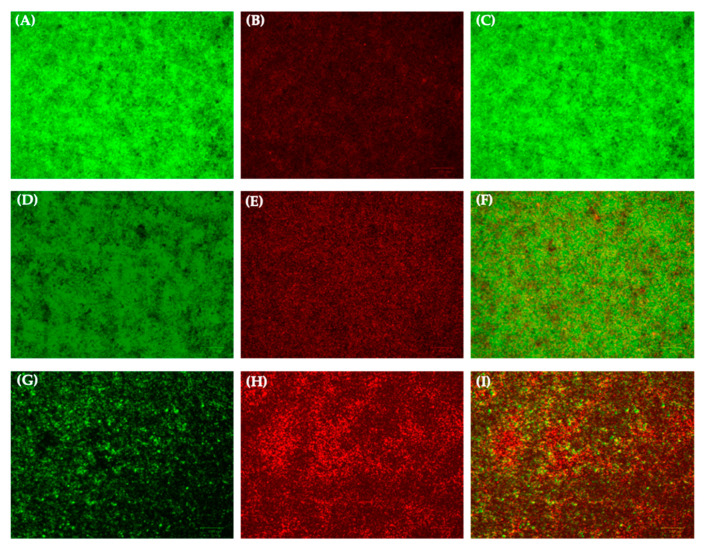
Images of *S. aureus* ATCC 25923 biofilm, stained with LIVE/DEAD Kit, acquired via fluorescence microscopy: (**A**–**C**) untreated sessile population (CTR−); (**D**–**F**) bacteria exposed to 12.5 μM of Oreoch-1; (**G**–**I**) bacteria exposed to 25 μM of Oreoch-1.

The impairment of *S. aureus* biofilm under Oreoch-1 treatment was qualitatively corroborated by fluorescence microscopy analysis with LIVE/DEAD staining. PI penetration occurs only in membrane-damaged/dead bacterial cells, thus releasing red fluorescence; by contrast, SYTO-9 enters all the cells and accounts for green fluorescence [20]. As shown in Figure 5, the biofilm matrix challenged with 12.5 and 25 μM (D–F and G–I, respectively) exhibited less structural density and numerous dead cells associated with a more intense red staining as the peptide concentration increased, confirming a dose-dependent response. Contrarily, the untreated sessile population formed a homogeneous matrix, with few detectable red cells (A–C). 

## 4. Discussion

The drug resistance phenomenon is a major hurdle for the treatment of several infectious diseases, increasing the economic burden, prolonging hospitalization, leading to recurrent infections, and increasing fatalities [21]. Tuon et al. estimate that approximately 80% of chronic infections in humans are associated with biofilm formation [22]. Indeed, bacterial colonization and the further potential switch from a metabolically active to inactive, nondividing state (named a persister) within the biofilm are important causes of treatment failure. Conventional antibiotics target metabolically active bacteria and are ineffective against persisters, protected by the exopolysaccharide matrix from lethal concentrations of drugs and immune cells [23]. Hence, intensive scientific efforts have been devoted to the development of antimicrobial compounds with different mechanisms of action [14]. Contextually, more than 3000 AMPs have been reported to elicit activity against bacteria in their planktonic as well as sessile lifecycle so far [24,25]. In contrast to the single target of classic antibiotics, AMPs can exert their antibacterial activity impairing the cell membrane structure, permeability, and fluidity, and/or interfering with nucleic acids and protein synthesis pathways, triggering stress and ROS accumulation [26,27]. Thanks to their multihit, broad-spectrum action, AMPs may also provide a potential new therapeutic solution against MDR strains, supporting and enhancing the effectiveness of commercially available antibiotics [28]. For instance, Hylin-a1 peptide, derived from the skin of the frog *Heleioporus albopunctatus,* was recently reported by our group as a potent antimicrobial agent capable of inhibiting *S. aureus* invasion and modulating levels of inflammatory cytokines [16]. Furthermore, the interest in the identification and design of new molecules with greater potency, selectivity, and cost-effectiveness has been driven by the approval of some AMPs for human use [26,29,30,31]. Among Staphylococci, *S. aureus* is of most clinical concern because of its great biofilm-forming capacity on biomaterials and medical devices, the production of several toxins, and the ability to evolve resistance mechanisms [22,32]. On the other hand, antibiotic resistance is also a widespread property between *S. epidermidis* strains which, although generally considered less virulent than *S. aureus*, are frequently involved in nosocomial infections [14,33]. Biofilm production and the secretion of extracellular enzymes and toxins, together with the intracellular persistence ability, mainly account for *S. epidermidis* invasiveness [14]. In the present study, we highlighted the interesting in vitro antistaphylococcal properties of a piscidin-like AMP, Oreoch-1, providing an efficient way to counteract the suspended and the adherent biofilm forms of *S. aureus* and *S. epidermidis*. Oreoch-1, a 23 amino acid-long cationic peptide isolated from the gills of Nile tilapia (*Oreochromis niloticus*), is considered a fruitful source of bioactive compounds with antimicrobial, anticancer, and immunomodulatory activities [34,35,36,37]. Members of the piscidin family identified in fish immune cells have displayed interesting action in vivo and in vitro against human bacterial, fungal, and parasitic pathogens [38,39,40]. To explore its antibacterial spectrum, a C-terminally amidated Oreoch-1 peptide was assayed against standard, biofilm-positive, biofilm-negative, and MDR clinical strains of *S. aureus* and *S. epidermidis*. The peptide under analysis registered an interesting action against *S. aureus* strains at nontoxic concentrations (≤25 μM), with MIC_80_ values in the range 6.25–25 μM, as well as against *S. epidermidis* cells, with MIC_80_ values between 12.5 and 25 μM. On the other hand, the SA3 isolate showed less susceptibility to Oreoch-1, inhibiting the bacterial growth by only 66%, perhaps due to its isolation source (hemoculture) and its acquired invasiveness. Several studies have interestingly shed light on AMPs and their therapeutic potential against *S. epidermidis*, but to the best of our knowledge, this is the first study reporting the susceptibility of this opportunistic pathogen toward piscidins [14,41,42,43]. In agreement with our findings, Acosta et al. highlighted relevant anti-Gram-positive bacteria efficacy for Oreoch-1, recording MIC values of 5 and 3 μM against *S. aureus* and *Bacillus subtilis*, respectively, and a good cytotoxicity profile against human red blood cells [34]. Although the amphipathic α-helix conformation predicted by their in silico analysis suggested a bactericidal effect, directly destroying the target cell membrane, our results indicate a bacteriostatic action [34]. Huang et al. exploited a mouse model infected with methicillin-resistant *S. aureus* (MRSA) and treated with tilapia piscidin 3 (TP3), a peptide differing from Oreoch-1 for the position 18 (Ser18/Gly). They observed significantly enhanced animal survival, the control of bacterial burden with a bacteriostatic effect in infected tissues, and accelerated wound closure, when compared to the untreated mice [36]. In brief, the real mechanism of action of Oreoch-1 remains unknown and will be the subject of further study. *S. aureus* together with *S. epidermidis* causes around 2/3 of implant infections, requiring a high-dose and long-term antibiotic treatment, and in the most severe cases, surgical debridement [44]. Indeed, the biofilm associated with medical devices may pave the way for chronic and recalcitrant infections [21]. Currently, two main types of antibiofilm strategies have been displayed: inhibiting/preventing new biofilm formation and eradicating existing biofilms [22]. In this context, we explored the potential activity of Oreoch-1 against biofilm, an important feature of staphylococcal infections. The CV staining assay revealed a significant reduction in *S. aureus* biofilm formation at subinhibitory concentrations (1/2-1/8×MIC, *p*-value = 0.0001), whilst *S. epidermidis* sessile community development was significantly prevented only at 12.5 μM (*p*-value < 0.0248). The biofilm inhibitory properties on *S. aureus* were confirmed by the plate counting method and then by fluorescence microscopy analysis via the LIVE/DEAD staining kit, which showed a thin and irregular matrix. In this regard, Chang et al., challenging MRSA with tilapia piscidin 4 (TP4), observed MIC, minimum biofilm inhibitory concentration (MBIC), and minimum biofilm eradicating concentration (MBEC) values of 16, 8, and 256 μg/mL, respectively [45]. Interestingly, several studies have reported the use of AMPs in biomedical coatings, to avoid bacterial colonization and to support functionalities such as drug delivery and integration into the host [46,47,48]. 

## 5. Conclusions

In summary, as drug-resistant Staphylococci have relevantly challenged the available therapeutic options, the discovery of new antimicrobial molecules is mandatory [41]. Our results regarding the in vitro cytotoxic profile together with the interesting antibacterial and antibiofilm properties suggest that Oreoch-1 may be a potential new weapon against two staphylococcal species associated with potentially life-threatening infections. However, this study only provides a preliminary and in vitro analysis of the antimicrobial potential of this peptide. Further experiments are required to assess the safety profile of Oreoch-1 on animal models, which could strengthen its potential clinical use for the treatment of *Staphylococcus* infections.

## Figures and Tables

**Table 1:** Features of the staphylococcal strains used in the current study.

*Staphylococci*	Strain Code	Resistance Profile	Clinical Specimen
*S. aureus*	ATCC 6538	Standard strain	
*S. aureus*	ATCC 25923	Standard strain	
*S. epidermidis*	ATCC 12228	Standard strain	
*S. epidermidis*	ATCC 35984	Standard strain	
*S. aureus*	SA1	Methicillin-, macrolides-, fluoroquinolones-resistant	Nasal swab
*S. aureus*	SA2	Methicillin-susceptible, aminoglycoside-resistant, inducible MLSb phenotype	Pharyngeal swab
*S. aureus*	SA3	Methicillin-susceptible, tetracycline-resistant, inducible MLSb phenotype	Blood culture
*S. epidermidis*	SE1	Multi-sensitive	Venous Catheter
*S. epidermidis*	SE2	Methicillin-, fluoroquinolones-, aminoglycosides-, rifamicins-, tetracycline-resistant, inducible MLSb phenotype	Blood culture
*S. epidermidis*	SE3	Fluoroquinolones, lincosamides-, macrolides-, aminoglycosides-, rifamicins-, oxazolidinones-resistant	Blood culture

## Data Availability

The data presented in this study are available on request from the corresponding author. The authors can confirm that all relevant data are included in the article.
